# A Rare Complication of Oropharyngeal Tularemia: Dacryocystitis

**DOI:** 10.4274/tjo.galenos.2018.96337

**Published:** 2019-06-27

**Authors:** Helin Ceren Köse, Melek Banu Hoşal

**Affiliations:** 1 Ankara University Faculty of Medicine, Department of Ophthalmology, Ankara, Turkey

**Keywords:** Tularemia, nasolacrimal duct obstruction, dacryocystitis

## Abstract

Tularemia is a zoonotic disease caused by *Francisella tularensis*, a highly virulent gram-negative coccobacillus. Oropharyngeal tularemia, one of the clinical subtypes, is the most common clinical form of the disease in Eastern Europe, including Turkey. This clinical form affects mostly the head and neck region and the most common complaints of patients are mass in the neck, sore throat, and fever. This form of tularemia may be confused with tonsillitis, pharyngitis, or cervical lymphadenitis caused by other microbial agents due to the nonspecific clinical and laboratory features. In this study, we present a patient with nasolacrimal duct obstruction and dacryocystitis caused by oropharyngeal tularemia.

## Introduction

Tularemia is a zoonotic infection caused by *Francisella tularensis*, a highly virulent gram-negative coccobacillus. *F. tularensis* is endemic in the northern hemisphere, especially in Russia, Kazakhstan, Turkmenistan, some states of the USA, Canada, and some European countries such as Finland and Sweden.^1^ Due to its high virulence, it periodically causes epidemics in Turkey. Rodents such as rabbits, beavers, rats, and mice and mammals such as raccoons, cats, dogs, and cattle are the primary reservoirs of infection for humans.^2,3^ Studies have shown that the most common sources of transmission to humans are rodents such as field mice and house mice.^4,5^ Mosquitoes, horseflies, fleas, and lice act as vectors of *F. tularensis*. Farmers, hunters, and forest workers in endemic areas are groups at risk of infection. In humans, the infection usually occurs after contact with infected animals or through the bites of arthropod vectors. Other routes of transmission include contact with the body fluids of an infected person, consumption of contaminated food or drink, inhalation of contaminated aerosols, contact of these fluids or aerosols with the eye, or rubbing the eyes with contaminated fingers.^6^

Tularemia has six clinical subtypes: ulceroglandular, glandular, pneumonic, typhoidal, oculoglandular, and oropharyngeal. The oropharyngeal form is the most common clinical presentation in the Eastern European region, including Turkey.^7^ In this form, the disease is localized to the head and neck area and manifests with signs such as sore throat, fever, and neck mass.^8,9^

In this study, we discuss a patient who developed nasolacrimal duct obstruction and dacryocystitis associated with oropharyngeal tularemia.

## Case Report

**Figure 1:**
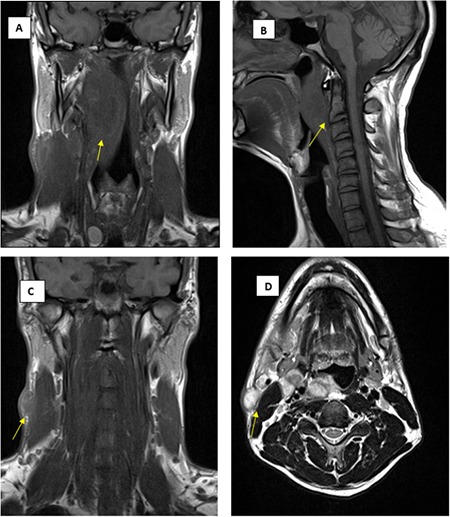
A,B) Magnetic resonance images of the retropharyngeal abscess occluding the right oropharynx (yellow arrows). C,D) Magnetic resonance images of lymphadenopathies including cystic and necrotic areas in the right submandibular region (yellow arrows)

A 33-year-old man presented to our clinic with complaints of watering, redness, and purulent discharge in the right eye. The patient reported seeing a physician a year earlier in Georgia due to fatigue, nausea, vomiting, and diarrhea. After his diarrhea and vomiting had resolved, he had swelling of the lymph nodes on the right side of the neck. After returning to Turkey for treatment, he had received cephalosporin and penicillin for suspected pharyngitis. When night sweating and weight loss were added to his complaints, he had presented to another hospital where his treatment was changed to amoxicillin-clavulanic acid 1 g 3 times a day and ciprofloxacin 750 mg twice a day, and incisional drainage was performed on the lymph nodes of his neck. When his symptoms failed to resolve completely, he had presented to the department of infectious diseases of a different university hospital. Serum agglutination test was positive for *F. tularensis* at a titer of 1/1280 and he was prescribed streptomycin 1 g per day for 9 days followed by 1 g twice a day for 5 days for a total of 14 days, followed by doxycycline 100 mg twice a day for 1 week. Ultrasound examination of the neck had revealed multiple abscesses in the right submandibular region and pathological lymph nodes including multiple calcifications in the right cervical chain, while magnetic resonance imaging of the neck showed retropharyngeal abscess narrowing right nasopharynx and oropharynx and submandibular lymphadenopathies (LAP) including cystic and necrotic areas (Figure 1). He reported that the LAPs had resolved after a few months with no recurrence, but complaints of watering, swelling in the lacrimal sac area, hyperemia, and pain in the right eye developed a few weeks later. The patient presented to our clinic with recurrent swelling around the lacrimal sac, hyperemia, and purulent discharge.

**Figure 2:**
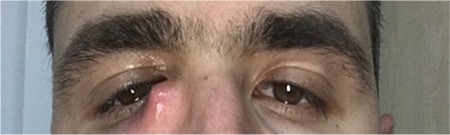
Clinical presentation of the patient with abscess in the nasolacrimal area

On examination his best corrected visual acuity was 20/20 in both eyes. Intraocular pressure measured by automatic tono-pneumometry was 15 mmHg in each eye. On slit-lamp examination, epiphora was noted in the right eye and the left eye was normal. There was swelling in the area of the right lacrimal sac (Figure 2). Fundus examination was normal in both eyes. In nasolacrimal lavage, the patient’s right nasolacrimal duct was occluded and the common canaliculus was patent. Discharge of purulent material from the right lower punctum was noted after lavage. A sample of the purulent discharge was collected and sent to the microbiology laboratory for culturing and the patient was started on oral amoxicillin-clavulanic acid 1 g twice a day and topical ciprofloxacin drops 4 times a day. Antibiotherapy was discontinued because the culture was negative. Consultation from the otorhinolaryngology (ENT) department was requested to rule out any intranasal pathology. The patient underwent ENT examination, followed by nasal endoscopic examination. In addition, to rule out intranasal pathologies that may present an obstacle to surgery, the paranasal sinuses were examined using computed tomography. No intranasal pathologies were detected in ENT evaluation. Dacryocystorhinostomy surgery was recommended to the patient, but he refused the procedure.

## Discussion

*F. tularensis* causes infections in humans after entering the body via direct inoculation to the skin or mucous membrane, inhalation of the bacteria, or consumption of contaminated water or food. Its incubation period varies between 1 and 14 days, though it usually appears 3-6 days after exposure. Although the symptoms of tularemia vary depending on the area of involvement, onset is usually characterized by fever, flu-like symptoms, and cervical LAP.^10,11^

Because *F. tularensis* is very small and stains weakly in Gram staining, direct detection in patient samples has no diagnostic value. A rich medium is required for its growth, and its high virulence and ability to spread via inhalation pose a risk for laboratory personnel. Therefore, routine isolation of the bacteria is not recommended.^12^ Microagglutination assay is a valuable serological diagnostic method, with a single titer ≥1/160 or rising titer being diagnostic. Antibodies against *F. tularensis *can be detected using tube agglutination, microagglutination, hemagglutination, and enzyme linked immunosorbent assay methods.^13^ Due to the difficulty of growing *F. tularensis* in culture and the late reporting of serological test results, research is ongoing to develop rapid diagnostic methods. Detection of antigens in urine, direct fluorescent antibody staining, and polymerase chain reaction (PCR) are some of these methods. The most commonly used and most advantageous of these methods is PCR.^12,13^

Ulceroglandular disease, which is the most common subtype of tularemia, is usually transmitted by tick bite.^10^ After an incubation period of 3-6 days, it manifests with signs such as flu-like symptoms, fever, headache, and fatigue. Local proliferation of the bacteria at the bite leads to the formation of papules and skin ulcers. The bacteria spread via the lymph system from the ulcer at the site of the tick bite to local lymph nodes.^11,14^ Similarly, the glandular form is also transmitted via arthropod vector but is not characterized by skin ulcers. Pneumonic disease is the most severe clinical form, presenting with symptoms such as dry cough, chest pain, and difficulty breathing.^15^ Typhoidal disease, which is a very rare form, manifests with fever, vomiting, diarrhea, splenomegaly, and hepatomegaly.^11^

In oculoglandular disease, a relatively rare form, the infectious agent is usually transmitted via rubbing the eyes with contaminated fingers or by contact of contaminated aerosols and fluids with the eye.^16^ Clinically, oculoglandular tularemia usually manifests as unilateral conjunctivitis and painful LAP. Oculoglandular disease accounts for approximately 3-5% of all cases. It can involve the eyes, eyelids, and more rarely, the lacrimal system.^16,17,18^ Lacrimal system involvement in oculoglandular tularemia was previously reported as purulent conjunctivitis and dacryocystitis in a 27-year-old woman who was 18 weeks pregnant.^19^ Considering the patient’s pregnancy, the disease was treated with topical gentamicin and oral amoxicillin-clavulanic acid therapy for 2 weeks, and clinical cure was achieved. The dacryocystitis was treated with surgical drainage, which was repeated a few weeks later due to relapse. After resolution of acute dacryocystitis, no further relapse was observed.

Oropharyngeal disease is the most common clinical form of the disease in the Eastern European region, including Turkey.^8^ This clinical form is localized to the head and neck area and manifests with signs such as sore throat, fever, and neck mass. The source of infection is usually contaminated water and food. Due to the nonspecific clinical and laboratory findings, it can be misdiagnosed as tonsillitis, pharyngitis, or cervical lymphadenitis associated with other microbial agents. In oropharyngeal tularemia, neutrophilic and granulomatous infiltration in the cervical lymph nodes leads to necrotizing lymphadenitis and abscess formation. The lymph nodes are filled with pus and may spontaneously rupture and drain to the skin. This suppuration may also continue after the initiation of antibiotic therapy. Bacteria can also be disseminated via the bloodstream to the spleen, liver, lungs, kidneys, colon, and skeletal muscles.^9,20^

The macrophage cell-mediated immune response plays a major role in the pathogenicity of *F. tularensis*, which is a facultative intracellular bacterium. When macrophages attempt to digest the bacterium, it escapes from the phagosome into the cytoplasm. Continuing to proliferate in the cytoplasm, it induces macrophage cell death, which enables the infection to spread.^21^ Similar mechanisms also apply to neutrophils. After phagocytosis, *F. tularensis* suppresses oxidative pathways by inhibiting nicotinamide adenine dinucleotide phosphate oxidase. The natural immune response that occurs as an early response to infection causes the release of proinflammatory cytokines such as interleukin 1, interleukin beta, and interleukin 18 from macrophages in the cytoplasm, which in turn induce caspase-1-dependent cell death. In this way, type-1 interferon is secreted. With CD4+ and CD8+ T-cell activation in response to protein antigens, macrophages can kill the bacteria via phagocytosis with the help of tumor necrosis factor-α, γ-interferon, and reactive oxygen species.^22,23^ Phenotypic characteristics of the bacteria such as lipopolysaccharides, type-4 pili, capsule, acid phosphatase enzyme, and siderophores are among its virulence factors.^24^

Paulsen et al.^25^reported that inflammation in the nasolacrimal duct can lead to the development of dacriostenosis. Inflammation triggers edema in the mucous membranes, remodeling of the helical structure of connective tissue fibrils, and disruption of subepithelial cavernous body function due to reactive hyperemia, resulting in temporary occlusion of the lacrimal system. Recurrent dacryocystitis episodes associated with this occlusion can affect the epithelial and subepithelial tissues, and a fibrous occlusion may develop in the lumen of the nasolacrimal duct.^25^

Lingberg and McCormick^26^ showed that inflammatory infiltrates and edema in the nasolacrimal duct lead to the development of chronic dacryocystitis. They reported that with prolonged inflammation in the nasolacrimal duct, the inflammatory process is replaced by fibrosis in the mid-term, and fibrous occlusion forms in the nasolacrimal duct in the long term.

Another recent finding about acquired nasolacrimal duct occlusion is the mucosa-associated lymphoid tissue surrounding the lacrimal sac and duct. It is believed that these local lymphoid structures, called lacrimal drainage-associated lymphoid tissue, are involved in immune modulation and that damage to these structures may lead to the development of nasolacrimal duct occlusion.^27,28^

We believe that in our patient, oropharyngeal tularemia infection spread to the mucosa-associated lymphoid tissue around the nasolacrimal duct via the lymphatic system through the cervical lymph nodes or via local adjacency caused by inflammation in the area, and this inflammation led to the development of edema in the mucous membranes and later to fibrosis development in the nasolacrimal duct. Occlusion of the lacrimal system due to fibrosis manifested with recurrent episodes of dacryocystitis.

In conclusion, any infectious or inflammatory event within the nasal cavity may lead to the development of nasolacrimal duct occlusion, especially in individuals with predisposition due to anatomic factors. Nasolacrimal duct occlusion and associated dacryocystitis may develop as a rare complication of oropharyngeal tularemia.
